# Sputum Smear Non-conversion at the End of Intensive Phase of Tuberculosis Treatment at a Tertiary Care Center in Nepal: A Descriptive Cross-sectional study

**DOI:** 10.31729/jnma.7020

**Published:** 2021-11-30

**Authors:** Naveen Prakash Shah, Anil Regmi, Aakash Acharya, K.C. Jwala, Bidur Khatiwada, Meera Hada

**Affiliations:** 1 Department of Pulmonology, National Tuberculosis Control Center, Sanothimi, Bhaktapur, Nepal; 2 Department of Reference Lab, National Tuberculosis Control Center, Sanothimi, Bhaktapur, Nepal

**Keywords:** *antitubercular agents*, *mycobacterium tuberculosis*, *tuberculosis*

## Abstract

**Introduction:**

Sputum non-conversion is smear positive tuberculosis despite anti-tubercular therapy. Various factors may lead to sputum non-conversion including resistance to anti-tubercular drugs, age, gender, disease severity, non-compliance, drugs unavailability etc. Little is known and studied about the contribution of these individual factors. Our study sought to determine the prevalence of sputum smear non-conversion in patients at the end of intensive phase of tuberculosis treatment visiting a tertiary-level institution in Nepal.

**Methods:**

A descriptive cross-sectional study was conducted among recorded data of patients undergoing sputum Acid Fast Bacilli staining at the end of intensive phase at National Tuberculosis Control Center from April 2018 to April 2020. The study was approved by Nepal Health Research Council (Registration no: 76012020 P). The convenient sampling method was adopted. The data were analyzed using Microsoft Excel. Point estimate at 95% Confidence Interval was calculated along with frequency and proportion for binary data.

**Results:**

Our study found that out of 830 samples that were tested by Acid Fast Bacilli stain at the end of intensive phase, 40 (4.82%) (3.37-6.28 at 95% Confidence Interval) were sputum smear nonconverters. The mean age of sputum non-converters was 41.25+15.543 years.

**Conclusions:**

The study shows that a significant proportion of patients remain acid-fast stain positive despite the treatment. However, the proportion is low compared to other similar studies around the globe. This study provides program managers with evidence to support the development of more tailored tuberculosis care and need to conduct more intensive studies about various factors that may lead to non-conversion.

## INTRODUCTION

Tuberculosis (TB) is an airborne infection caused by Mycobacterium tuberculosis.^1,2^ In 2018, TB affected around 10,00,000 people worldwide while 69,000 people in Nepal.^3,4^ AFB stain is used for the diagnosis of TB.^5,6^ Sputum AFB non-conversion are cases of pulmonary TB who remain AFB positive despite anti-tubercular therapy (ATT).^7^ Various factors may contribute to development of non-conversion which include resistance to anti TB drugs, age, gender, disease severity, non-compliance, drugs unavailability, etc.^8,9^

Sputum non-conversion rates have been increasingly observed during the management of TB patients.^10^ No significant study has been performed in recent years in Nepal to find out the prevalence of sputum non-conversion in patients undergoing anti-tubercular therapy and to stratify the role of various causes that might have contributed to the development of nonconversion.

This study aims to find out the prevalence of sputum smear non-conversion at the end of the intensive phase of TB treatment at a tertiary center in Nepal.

## METHODS

This descriptive cross-sectional study was conducted in the National Tuberculosis Control Center in Nepal from April 2018 to April 2020 using the data from medical records. The study was approved by Nepal Health Research Council (reference number: 76012020 P). Convenient sampling was done and the sample size was calculated as follows,

n = Z^2^ × p × q / e^2^

  = (1.96)^2^ × 0.5 × (1-0.5) / (0.04)^2^

  = 600

Where,

n= minimum required sample size,
Z= 1.96 at 95% Confidence Interval (CI),
p= prevalence taken as 50% for maximum sample size,
q= 1-p,
e= margin of error, 4%

The sample size was calculated to be 600. However, we took data from 830 patients. All new cases of sputum positive pulmonary TB within 2 years were included in the study. Sputum AFB negative cases for suspected pulmonary TB, clinically diagnosed PTB cases, previously treated PTB cases, cases of pulmonary TB diagnosed for the first time by Gene Xpert test or sputum culture, extra pulmonary TB cases with no primary involvement of lungs, and sputum smear positive but with incomplete data in database were excluded from the study.

The data were entered and analyzed in Microsoft Excel. Point estimate at 95% CI was calculated along with frequency and percentage for binary data.

## RESULTS

**Figure 1:**
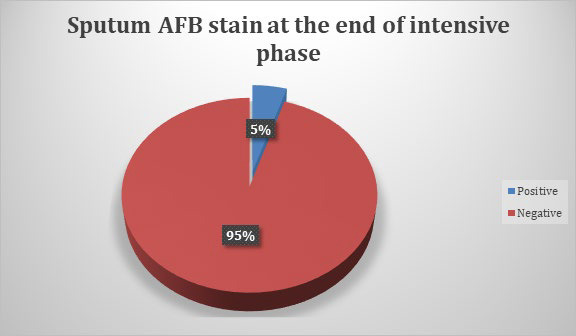
Sputum AFB stain at the end of intensive phase.

Our study concluded that out of 830 samples that were tested for M. tuberculosis at the end of intensive phase, 40 (4.82%) (3.37-6.28 at 95% Confidence Interval) were sputum smear non-converters (Figure 1).

Out of 40 samples, mean age of sputum non-converters at the end of intensive phase was 41.25±15.543 years. In females, the mean age of sputum non-converters was 35.64±18.473 years and in male mean age was 43.38±14.055 years.

**Figure 2:**
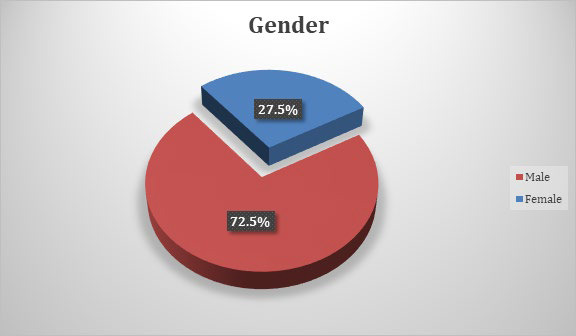
Gender distribution of sputum smear nonconversion.

29 (72.5%) males were found to be sputum nonconverters at the end of intensive phase compared to 11 (27.5%) females (Figure 2).

**Figure 3:**
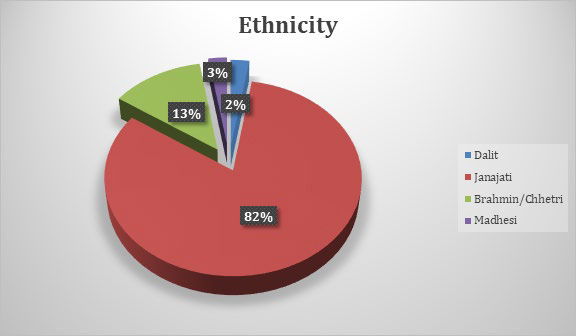
Sputum smear non-conversion based on ethnicity.

Sputum non-converters were most prevalent among Janajati communities (82%) followed by Brahmin/Chhetri (13%) (Figure 3).

## DISCUSSION

Various factors may lead to sputum smear nonconversion including drug resistance, non-compliance, availability of the drug in DOTS center, bacterial load at the start of treatment, disease severity at the start of treatment, comorbid conditions, malnutrition, smoking status, etc.^8,9^ The percentage of smear-positive patients with delayed sputum smear conversion at the end of the intensive phase of anti-TB treatment is an indicator of the performance of the TB programme.^10^ Thus, it is necessary to study the sputum non-conversion rates in different settings. In addition, a detailed study is necessary to be carried out to stratify the role of these factors.

Among the patients who visited NTCC between 14th April 2018 and 13th April 2020 and were found to be sputum smear-positive, 4.82% of patients were sputum smear non-converters following two months of initiation of ATT. It suggests that a significant proportion of patients who completed two months of intensive therapy are found to be non-converters. The findings of our study are supported by the findings of another study performed in Morocco which shows the respective prevalence to be 5%.^11^ However, the rate is low compared to 13.3% in Nigeria, 15.7% in Uganda, and 15% in Ethiopia.^12-14^ Lower rates of nonconversion in our study may be because of several reasons. African countries have a higher incidence of TB as well as higher drug resistance rates resulting in a higher percentage of non-converters.^15-16^ Poverty, malnutrition, and drug unavailability could also be other factors.^16^ At the same time, underreporting and lack of documentation could have shown a lower rate of non-converters in our study. Non-compliant patients not visiting the DOTS center for sputum tests following intensive therapy could also be another contributing factor. In addition, among the nonconverters, 72.5% were male and 80% belonged to the Janajati community.

Despite universal direction and national plans for TB treatment, control of TB has been a global challenge.^3^ TB epidemiology and resistance patterns could have changed in recent years needing different screening and rapid DST approaches.^17^ This study provided valuable insight regarding the non-conversion rates among new cases, which might be important in exploring this in detail with another survey.

Since our study is limited to NTCC only, the findings cannot be generalized. Likewise, a low incidence rate compared to other countries where TB is endemic may suggest that it is necessary to conduct a large-scale study to find out the actual information. We have not considered the clinical improvement in the study. Likewise, comorbid conditions and associated risk behavior (HIV, DM, smoking status, alcohol intake, overcrowding) were not assessed and cases were not followed for sputum culture. Sample contamination and record mismatch are also a matter of concern along with limited resources and budget.

## CONCLUSIONS

The study shows that a significant number of patients were found to be sputum smear non-converters following two months of anti-tubercular therapy and it supports the need of conduction of other studies to accurately quantify the rate of non-conversion at the end of intensive phase of ATT. It urges the members of the National TB plan to further conduct national level studies to find out the actual data regarding nonconversion rates as well as effects of various factors that may lead to sputum non-conversion in Nepalese population. The data may then help to plan the approach to management of TB patients in Nepal.
